# Epidemiology of Antimicrobial Residues and Phenotypic Resistance of Bacterial Isolates from Waste Milk on California Dairies

**DOI:** 10.3390/microorganisms14030620

**Published:** 2026-03-10

**Authors:** Yotam Mihreteab, Emmanuel Okello, Pramod Pandey, Essam Abdelfattah, Pius S. Ekong, David Sheedy, Wagdy R. ElAshmawy, Betsy M. Karle, Randi A. Black, Deniece R. Williams, Sharif S. Aly

**Affiliations:** 1 Veterinary Medicine Teaching and Research Center, School of Veterinary Medicine, University of California Davis, Tulare, CA 93274, USA; ymmihreteab@ucdavis.edu (Y.M.); eokello@ucdavis.edu (E.O.); eabdelfattah@ucdavis.edu (E.A.); pekong@ucdavis.edu (P.S.E.); david.sheedy@sydney.edu.au (D.S.); welashmawy@ucdavis.edu (W.R.E.); dvmwilliams@ucdavis.edu (D.R.W.); 2 Department of Population Health and Reproduction, School of Veterinary Medicine, University of California, Davis, CA 95616, USA; pkpandey@ucdavis.edu; 3 Department of Animal Hygiene and Veterinary Management, Faculty of Veterinary Medicine, Benha University, Moshtohor 13736, Al Qalyubiyah, Egypt; 4 Department of Internal Medicine and Infectious Diseases, Faculty of Veterinary Medicine, Cairo University, Giza 12211, Egypt; 5 Cooperative Extension, Division of Agriculture and Natural Resources, University of California, Orland, CA 95963, USA; bmkarle@ucanr.edu; 6 Cooperative Extension, Division of Agriculture and Natural Resources, University of California, Santa Rosa, CA 95403, USA; rablack@ucanr.edu

**Keywords:** antimicrobial resistance, accelerated failure time model, MIC, non-saleable milk, maximum residue limit

## Abstract

Waste milk (WM) on dairies is commonly fed to pre-weaned calves, raising concerns about antimicrobial drug (AMD) residues and their potential role in selecting for antimicrobial-resistant bacteria in their gut microbiota. The current observational study assessed AMD residue prevalence in WM and examined associations with AMR patterns in its bacterial isolates. Over a 10-month period, 40 WM samples were collected from eight dairies across Northern California, Northern San Joaquin Valley, and Greater Southern California. ELISA was used to detect six AMD residues, and bacterial isolates (*n* = 348), including coliforms, *Streptococcus* spp., *Staphylococcus* spp., and *Staphylococcus aureus,* were tested for AMR. Antimicrobial resistance was evaluated using the broth microdilution test, and associations with the presence of residues were analyzed via interval-censored accelerated failure time models. Ceftiofur was the most frequently detected residue (30%), followed by penicillin (5%), florfenicol (5%), and sulfadimethoxine (5%). Resistance varied by bacterial species, with significant associations observed between florfenicol residues and resistance in coliforms (MIC ratio = 2.12; *p* < 0.01), and between ceftiofur residues and resistance in *Streptococcus* spp. (MIC ratio = 10.51; *p* = 0.03). These findings suggest that WM may contain low-level AMD residues linked to elevated AMR, highlighting the need for targeted antimicrobial stewardship practices to mitigate AMR dissemination in dairy calves.

## 1. Introduction

Antimicrobial drugs (AMDs) include natural, semi-synthetic, and synthetic compounds with antimicrobial activity and are commonly administered through oral, parenteral, or topical routes to combat bacterial, viral, or fungal infections [1,2,3]. Within the dairy industry, AMD are routinely employed to treat various cattle diseases, including mastitis, metritis, pneumonia, and diarrhea [1,4,5,6]. Bovine mastitis, the most economically significant disease in dairy production, is primarily caused by contagious pathogens such as *Staphylococcus aureus* and *Streptococcus agalactiae*, as well as environmental organisms including *Escherichia coli*, *Klebsiella* spp., and environmental streptococci [7]. In the dairy industry, mastitis infections often require antimicrobial treatment, typically via intramammary infusion [8,9]. Data from the USDA National Animal Health Monitoring System (NAHMS) Dairy 2014 study [10,11] reported that 85.6% of cows with mastitis received AMD treatment. At the operation level, 91.3% of dairy operations reported administering AMD that required a withdrawal period [10,11]. Additionally, cephalosporins were reported as the most commonly used class of AMD for the treatment of mastitis (63.2%), lameness (61%), reproductive disease (54.1%), and respiratory disease (54.1%) [10,11].

Regulatory agencies, such as the U.S. Food and Drug Administration (FDA), the European Food Safety Authority (EFSA), and Codex Alimentarius, define residues as pharmacologically active compounds, including parent drugs, metabolites, and degradation products, and have established maximum residue limits (MRLs) in milk and other food products to protect consumer health [2,6,12]. To prevent residue violations, milk harvested from treated cows may be subject to withholding by being segregated from the saleable milk. Similarly, milk from recently calved cows, known as transition milk, is withheld while the cows transition from colostrum to milk production and the withdrawal period from any dry-off treatment elapses. The non-saleable milk from these sources is commonly referred to as waste milk (WM). Concentration of AMD residues in WM varies depending on several factors, including the type and dosages of AMD administered as well as the amount of milk produced [1,2,13].

Despite being unsalable, WM serves as a common source of nutrition for pre-weaned calves on one-third of dairy farms in the United States [14,15,16,17,18,19]. To reduce economic losses, WM is commonly pasteurized and fed to calves [4,16,20,21,22,23] and has been associated with higher weight gain in calves compared to milk replacers [4]. However, the presence of AMD residues in WM may exert selective pressure within the calves’ gut microbiota, promoting the emergence of antimicrobial-resistant bacteria [16,20,24,25,26]. A randomized controlled trial conducted in Germany reported that calves fed WM showed elevated resistance levels in coliforms, *Staphylococcus* spp., and *Streptococcus* spp. as compared with calves fed bulk tank milk [21]. The European Food Safety Authority (EFSA) Panel on Biological Hazards (BIOHAZ) has also emphasized the risk posed by the consumption of WM by calves, as studies have shown selection for resistance typically occurring at sub-inhibitory concentrations [1,19,24,26,27,28,29]. Therefore, examining AMD residues in WM and their association with antimicrobial resistance (AMR) in bacteria, including coliforms, *Streptococcus* spp., *Staphylococcus* spp., and *S. aureus*, is essential for guiding WM management practices that protect calf and herd health, sustain dairy productivity, and mitigate the risk of AMR emergence and spread. The present study aimed to estimate the prevalence of residues of six commonly used AMDs, including ceftiofur, florfenicol, penicillin, tetracycline, tilmicosin, and sulfadimethoxine, in WM samples and to examine their association with AMR. We hypothesized that the presence and concentration of AMD residues in WM would be associated with phenotypic resistance to antimicrobials of the corresponding class among bacterial isolates.

## 2. Materials and Methods

### 2.1. Study Herds and Sample Collection

**Figure 1:**
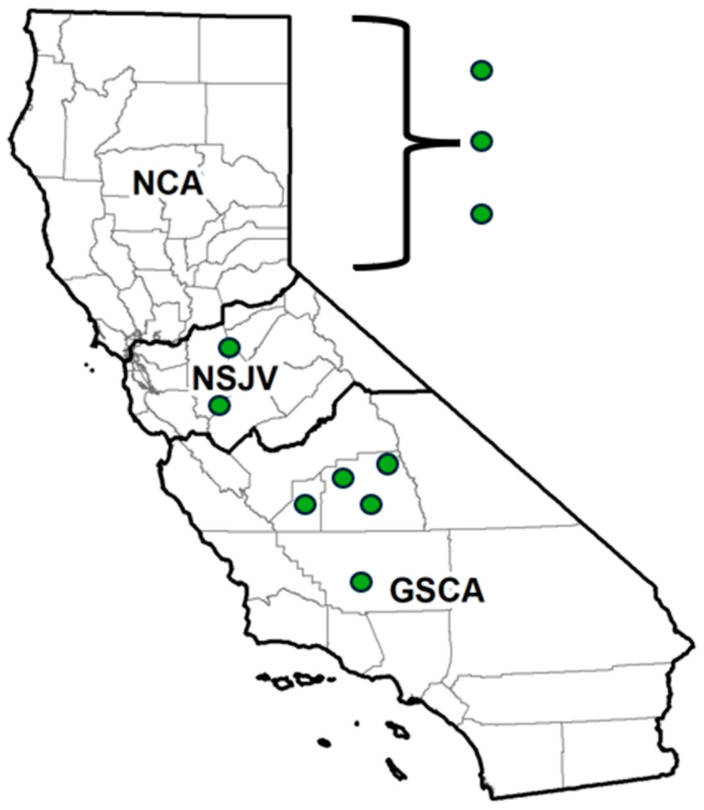
Geographic distribution of sampled dairies for antimicrobial drug (AMD) residue and susceptibility testing across California regions. Dairies located in the Northern San Joaquin Valley (NSJV) and Greater Southern California (GSCA) represent five of the ten enrolled operations where waste milk samples were collected for AMD residue detection between October 2018 and August 2019. For confidentiality reasons, the locations of the remaining three dairies in Northern California (NCA) are not disclosed. Two farms from the GSCA region were excluded from the AMD residue analysis.

Previously untested WM specimens from a longitudinal study on the epidemiology of AMR on California dairies and data on the respective dairies’ management and AMD use were utilized for the current study [15,30]. Of the 10 original study dairies, WM specimens and their metadata from 8 dairies were identified across the three distinct dairy-producing regions of California: Northern California (NCA), Northern San Joaquin Valley (NSJV), and Greater Southern California (GSCA) (Figure 1) [15,30]. These regions differ in infrastructure, herd management practices, and climatic conditions [30]. The participating herds were selected to ensure regional representation. NSJV included the original two farms in that region (herds 6 and 7 in the original study), GSCA included three farms (herds 1, 4, and 5) after excluding herds 2 and 3 due to budgetary reasons, and NCA included the three original farms in that region (herds 8, 9, and 10) [15]. To maintain confidentiality, specific county information for the NCA farms was withheld; however, the participating farms in GSCA and NSJV were in Kern, Tulare, Kings, and Stanislaus counties.

Sampling was designed to capture seasonal variability over a 10-month period spanning two seasonal cohorts: the fall–winter cohort (October 2018–February 2019) and the spring–summer cohort (March–August 2019). Due to budgetary reasons, WM samples from five of the original study’s 10 predefined sampling points were selected for testing while maintaining their distribution across the original study’s seasons: October–November (month 1), December–January (month 3), February–March (month 5), April–June (month 7), and July–August (month 10). These sentinel sampling points were chosen to maintain coverage of both seasonal cohorts and capture transition and peak periods, where mastitis incidence, AMD use, and hospital-pen WM composition, and therefore, AMD residues and AMR selection pressure are most likely to vary. Samples were collected directly from WM storage tanks in the hospital pens at each farm. At each sampling point, 50 mL of WM was collected aseptically into sterile polypropylene sample tubes (Capitol Vial Inc., Auburn, AL, USA). Samples were immediately placed on wet ice and transported to the Dairy Epidemiology Laboratory at the UC Davis Veterinary Medicine Teaching and Research Center (VMTRC, Tulare, CA, USA).

**Figure 2:**
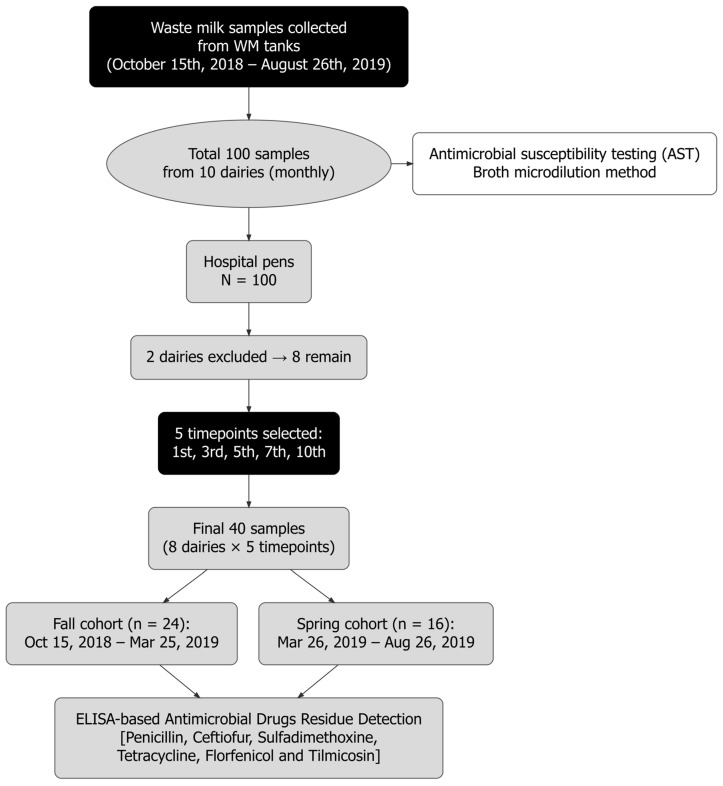
Flow diagram summarizing the number of WM samples collected and assessed for antimicrobial drug (AMD) residues and antimicrobial resistance in bacteria isolated from waste milk collected from hospital-pen samples during the fall–winter and spring–summer cohorts on 8 California dairies.

Upon arrival at the laboratory, 50 mL aliquots of fresh WM were processed for bacterial culture, and the remaining sample volume was stored at − 80 °C until AMD residue analysis. Over the study period, a total of 100 WM samples were collected. All 100 samples were subjected to bacterial isolation and AMR testing to compare AMR patterns across farms, regions, and seasons. Of these 100 samples, 40 were systematically selected through stratification by sampling month for AMD residue quantification, targeting ceftiofur, florfenicol, tilmicosin, penicillin, tetracycline, and sulfadimethoxine (Figure 2).

### 2.2. Bacterial Isolation and Identification

Bovine Blood Agar (BBA) was used to culture bacteria from WM samples. The WM samples were streaked onto BBA plates and incubated aerobically at 37 °C for 18 h. Bacterial species recovered from the culture media were identified based on colony morphology, hemolytic patterns, and Gram stain reaction, as well as coagulase and catalase test results [13,28]. The primary genera of bacteria included coliforms, *Staphylococcus* spp., *Streptococcus* spp., and *S. aureus*. To confirm preliminary culture-based identification, additional bench tests were performed. These included a positive CAMP test for *Streptococcus* spp., using a washed cow esculin and ferric-citrate-based medium to confirm the isolation of Group B beta-hemolytic streptococci. A 3% KOH test was used to identify Gram-negative bacteria, and a coagulase-positive test with coagulase plasma containing EDTA was used to identify *S. aureus* from the *Staphylococcus* spp. Final identification of the bacterial species was done by 16S rRNA sequencing (UC Berkeley, Berkeley, CA, USA). From the 100 WM samples collected during the original study, a total of 381 bacterial isolates representing eight distinct bacterial species were obtained. Out of these, 348 isolates representing four bacterial species were chosen for AMR testing.

### 2.3. Antimicrobial Resistance of Bacterial Isolates from Waste Milk

Antimicrobial susceptibility testing (AST) was performed using the broth microdilution method [15,31]. The Sensititre^TM^ system (Thermo Fisher Scientific Inc., Waltham, MA, USA) was used to determine the minimum inhibitory concentration (MIC) for each isolate against the AMD included in the Sensititre^TM^ BOPO7F Vet Antimicrobial Susceptibility Testing plate (Thermo Scientific, Remel Inc., Lenexa, KS, USA) [15]. The BOPO7F plate was selected because it contains a broad spectrum of AMD commonly used in the treatment of mastitis and bovine respiratory diseases in cattle. Briefly, the AST procedure for each isolate was performed as follows: Starting with a fresh overnight colony on BBA plates, 3 to 5 bacterial colonies were suspended in sterile demineralized water (Thermo Scientific, Remel Inc., Lenexa, KS, USA). The bacterial suspension was then adjusted to an optical density (OD) of 0.5 McFarland turbidity standard using a Nephelometer. Following standardization, 10 μL of coliforms, *Streptococcus* spp., and *S. aureus*, and 30 μL of *Staphylococcus* spp. suspension was mixed into 11 mL of Mueller–Hinton Broth (MHB) (Thermo Scientific, Remel Inc., Lenexa, KS, USA) to further dilute and prepare the inoculum. Subsequently, 50 μL of the prepared inoculum was dispensed into each well of the 96-well BOPO7F Vet plate using the Sensititre^TM^ Automated Inoculation Delivery System (Thermo Scientific, Remel Inc., Lenexa, KS, USA). After inoculation, the plates were sealed and incubated at 34–36 °C for 18 to 24 h.

Following incubation, MIC values were read using the Sensititre^TM^ Vizion Digital MIC Viewing System for pinpoint growth in each plate well and analyzed with the Thermo Scientific SensititreTM SWIN Software System Version 3.3 (Thermo Scientific, Remel Inc., Lenexa, KS, USA). The MIC was defined as the lowest concentration of an AMD that inhibits visible bacterial growth. Quality control for MIC plates was conducted using five reference control strains through *E. coli ATCC 35218*, *E. coli ATCC 25922*, *Enterococcus faecalis ATCC 29212*, *Strep. pneumoniae ATCC 49619*, *and Histophilus somni 700025*, while purity and dilution accuracy were confirmed by streaking 10 μL of the positive samples on Bovine Blood Agar (BBA) [14,30]. Furthermore, during the study, routine use of ATCC quality control strains was implemented to ensure accuracy and consistency. The BOPO7F plate comprises 19 AMD: ampicillin, clindamycin, danofloxacin, enrofloxacin, florfenicol, gamithromycin, gentamicin, neomycin, penicillin, sulfadimethoxine, spectinomycin, trimethoprim–sulfamethoxazole, tetracycline, tiamulin, tilmicosin, tildipirosin, tulathromycin, tylosin, and ceftiofur. Based on cross-resistance profiles with the six AMD targeted for residue testing, we excluded gentamicin, neomycin, spectinomycin, danofloxacin, and enrofloxacin from analysis, as these AMD do not exhibit inter- or intra-class resistance relationships with the six AMD selected for residue detection [31,32,33]. Interpretation of AMR was based on MIC breakpoints established by the Clinical and Laboratory Standards Institute (CLSI) documents M100, VET01-4th Edition, and VET08, where available [34,35]. For AMD lacking CLSI breakpoints, interpretive criteria were adopted from relevant peer-reviewed articles [14]. Bacterial isolates were categorized as either susceptible or resistant, with intermediate results classified as resistant for analysis purposes.

### 2.4. ELISA Test for AMD Residues

Following collection, each WM sample was aliquoted into five 2 mL tubes to facilitate laboratory processing and stored at −80 °C. Quantitative analysis of AMD residues in the WM samples was conducted using an indirect competitive enzyme-linked immunosorbent assay (ELISA) targeting six key AMD residues: ceftiofur, florfenicol, tilmicosin, penicillin, tetracycline, and sulfadimethoxine. The following ELISA kits (Creative Diagnostics, Shirley, NY, USA) were used: DEIABL-QB56, DEIA039, DEIA038, DEIABL-QB25, DEIA046, and DEIABL-QB9. For each AMD, WM samples were processed following an optimized extraction protocol to maximize detection efficiency, in accordance with the manufacturer’s instructions. Before analysis, all reagents and microwell plates were equilibrated to room temperature (20–25 °C) for at least 30 min. For extraction, 20 μL of each milk sample was transferred into a 2 mL polystyrene centrifuge tube, followed by the addition of an AMD-specific extraction solution. The mixture was vortexed for 1 min to ensure homogeneity before further processing. During extraction, 1% salactin acid (Creative Diagnostics, Shirley, NY, USA) was used for tetracycline and florfenicol. Following extraction, 50 μL of the supernatant from each sample was added to each well of the ELISA plate. All standards and spiked controls were run in duplicate as part of the assay so that samples with concentrations lower than the kit detection limit cannot be missed. Absorbance was measured at 450/630 nm within 5 min after stopping the reaction using the stop solution (Creative Diagnostics, Shirley, NY, USA) and tested by ELISA.

The cutoff values used to interpret the ELISA results were as follows: penicillin: 2 ppb, tetracycline: 3 ppb, tilmicosin: 5 ppb, florfenicol: 0.5 ppb, ceftiofur: 5 ppb, and sulfonamides: 5 ppb in Supplementary Materials [32,33]. Samples with residue concentrations equal to or exceeding the detection limit were classified as detected for that specific AMD. Furthermore, FDA maximum residue limits (MRL) for milk used for human consumption were adopted for the WM samples. MRL is the legally permissible level of veterinary AMD residue in milk for human consumption. The FDA MRL for the tested AMD residues were: ceftiofur: 5 ppb, tetracycline: 300 ppb, sulfadimethoxine: 10 ppb, and florfenicol: 0 ppb, based on publicly available sources. The tolerance level for tilmicosin has not been established [34,35,36,37]. Finally, we applied a combined metric, where samples with concentrations at or above the kit detection limit were subsequently evaluated against the FDA MRL thresholds.

### 2.5. Statistical Analysis

Data on antimicrobial residues and AMR testing were compiled using Microsoft Excel (Microsoft Corp., Redmond, WA, USA) and then merged with sample collection metadata in a relational database using Microsoft Access (Microsoft Corp., Redmond, WA, USA). Statistical analyses were performed in Stata version 18 (Stata Corp LLC, College Station, TX, USA). Since all variables were categorical, results were summarized as proportions with their corresponding logit-based standard errors (SE) and 95% confidence intervals (CI) given the small sample size. Proportions were calculated for each AMD across season, region, and sampling month.

#### Interval-Censored Accelerated Failure Time (AFT) Model Specification

The association between AMD residues and AMR across bacterial species was evaluated using parametric interval-censored accelerated failure time (AFT) models for each AMD tested. The AFT model is a parametric survival model in which the dependent variable, typically representing time-to-event, was adapted to our study such that the minimum inhibitory concentration (MIC) values were classified as left-censored (≤MIC_min_), right-censored (>MIC_max_), or interval-censored (between two adjacent tested concentrations) [38,39]. Here, MIC_min_ and MIC_max_ represent the lowest and highest AMD concentrations assessed, respectively. All left-censored MIC values were assigned to a lower bound of zero, as negative MIC values are biologically implausible. In this context, the AFT model estimates a MIC ratio (analogous to the time ratio in survival analysis), representing the ratio of MIC associated with AMD residue exposure to that associated with non-exposure to the same AMD. For example, a MIC ratio of 1.5 indicates that the presence of a drug’s residue was associated with 50% higher MIC, compared to the MIC in samples without the residue [38,39]. For the study models, the outcome was the interval-censored MIC data, while the main predictor was the presence of AMD residues, with separate models for each of the four bacterial species. To account for the repeated measurements across five defined sampling points, models incorporated robust standard error estimates with dairy as the cluster. This approach relaxed the assumption of independence between observations and accounted for intra-group correlation [40]. For each bacterial species and AMD residue, both Weibull and log-logistic parametric distributions were evaluated to identify the best-fitting model. Model selection was guided by the Akaike Information Criterion (AIC), with lower values indicating better model fit. The log-logistic model was selected for the final analysis as it provided the best model fit. Potential confounders, including season, region, and sampling point, were evaluated alongside the exposure of interest, AMD residue. Confounding was assessed using the method of change-in-estimate, in which variables producing a ≥20% change in the MIC ratio were retained in the final model [41]. Model building followed a manual forward selection process, beginning with a univariate model including only the AMD residue, then sequentially adding potential covariates. Two-way interactions were subsequently evaluated both between covariates and the exposure of interest, as well as among covariates themselves, with variables retained if they improved model fit or satisfied confounding criteria. Tilmicosin and tetracycline were excluded from all analyses due to the absence of detectable AMD residues. The final multivariable model for each AMD was selected based on model goodness of fit after adjusting for confounding and testing for biologically important two-way interactions. A 5% significance level was applied in all hypothesis testing.

## 3. Results

### 3.1. Descriptive Results of AMD Residues

The results of AMD residues in WM samples using ELISA are shown in Table 1. Prevalence of the AMD residues was estimated using three cutoff threshold criteria: (i) the ELISA kit detection limit, (ii) FDA-established MRL, and (iii) a combined metric that constitutes a two-stage classification in which samples that were positive based on the kit detection limit were subsequently evaluated against FDA MRL. The combined metric was used to classify samples above the kit detection limit and exceeding the FDA-established MRL as detected. When evaluated using the ELISA kit detection limit, penicillin and ceftiofur were each positive in 62.5% of samples (95% CI: 46.1–76.5), followed by tetracycline at 40.0% (95% CI: 25.6–56.4), sulfadimethoxine at 22.5% (95% CI: 11.8–38.7), and florfenicol at 5.0% (95% CI: 1.2–18.9). Tilmicosin was not positive in any of the study samples. When evaluated against the FDA MRL, ceftiofur exceeded the MRL in 30.0% (95% CI: 17.5–46.5) of samples, and the lowest percentage was recorded for florfenicol, sulfadimethoxine, and penicillin at 5.0% each (95% CI: 1.2–18.9). Tetracycline never exceeded the FDA MRL; therefore, it was not considered for further analysis. Using the combined classification metric, which incorporated both ELISA kit detection capabilities and FDA MRL, ceftiofur remained the most frequently detected residue (62.5%; 95% CI: 46.1–76.5), followed by penicillin, florfenicol, and sulfadimethoxine at 5.0% each (95% CI: 1.2–18.9). Tetracycline and tilmicosin were not detected using this metric.

### 3.2. Descriptive Results of AMD Residues by Region, Season, and Sampling Month

**Figure 3:**
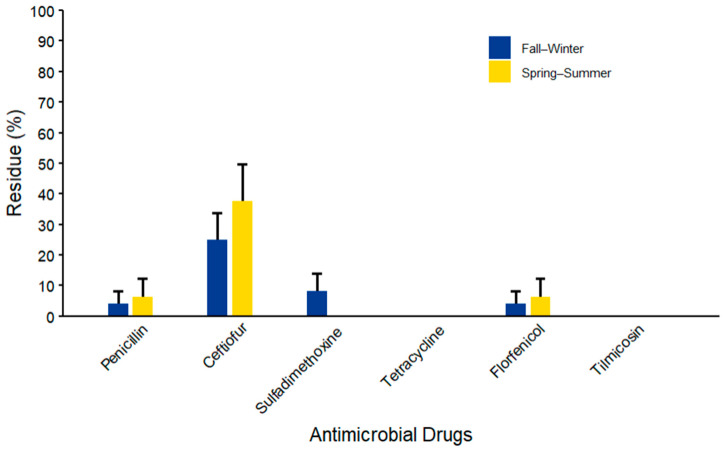
Percentage of antimicrobial drug (AMD) residues by season based on ELISA kit detection limits and FDA MRL thresholds in waste milk samples collected from 8 California dairies (*n* = 40). Error bars represent one standard error deviation from the mean.

**Figure 4:**
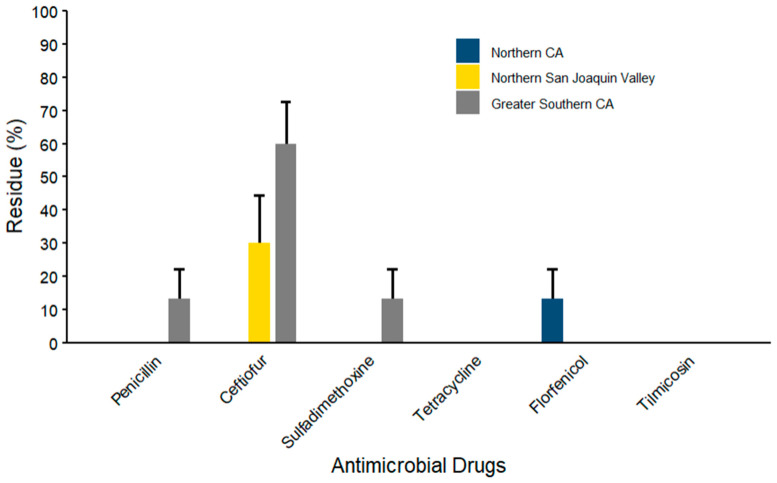
Percentage of positive antimicrobial drug (AMD) residue detection by region based on ELISA kit detection limits and FDA MRL thresholds in waste milk samples collected from 8 California dairy farms (*n* = 40). Error bars represent one standard error deviation from the mean.

The percentages of WM samples containing AMD residues, stratified by region and season, are summarized in Figure 3 and Figure 4 and Supplementary Material. Regarding regional variation, ceftiofur was detected based on the combined metric with a maximum detection frequency of 60.0% (*n* = 9/15; 95% CI: 34.1–81.3) in samples from the GSCA region. Penicillin and sulfadimethoxine were detected exclusively in GSCA, each at a frequency of 13.3% (*n* = 2/15; 95% CI: 3.2–41.7). Tetracycline residues detected in the WM samples were within the allowable FDA MRL, meaning it was considered negative based on the defined combined metric, while tilmicosin residues were not detected in any of the three surveyed regions. Florfenicol was the only AMD detected in NCA at 13.3% (*n* = 2/15; 95% CI: 3.2–41.7), while ceftiofur was the only AMD detected in NSJV at 30% (*n* =3; 95% CI: 9.6–63.4). Seasonal variation showed higher ceftiofur detection rates during spring-summer (37.5%, 95% CI: 17.4–63) as compared to the fall-winter (25%, 95% CI: 11.4–46.4) season (*n* = 16), whereas penicillin, sulfadimethoxine, and florfenicol had detection rates below 10% across both seasons. Results by sampling month are summarized in Supplementary Materials, Ceftiofur was the most frequently identified AMD, with detectable residues in all five sampling points, ranging from 12.5% (95% CI: 1.6–55.4) in October–November to 50% (95% CI: 19.3–80.7) in April–June. Sulfadimethoxine residues were observed only during October–November, with 25% (95% CI: 6.0–63.5) detection proportion in samples. Penicillin residues were detected in 12.5% of samples (95% CI: 1.6–55.4) during both February–March and April–June, while florfenicol was found at the same proportion in October–November and April–June.

### 3.3. Descriptive Results of AMR Among Isolates

**Figure 5:**
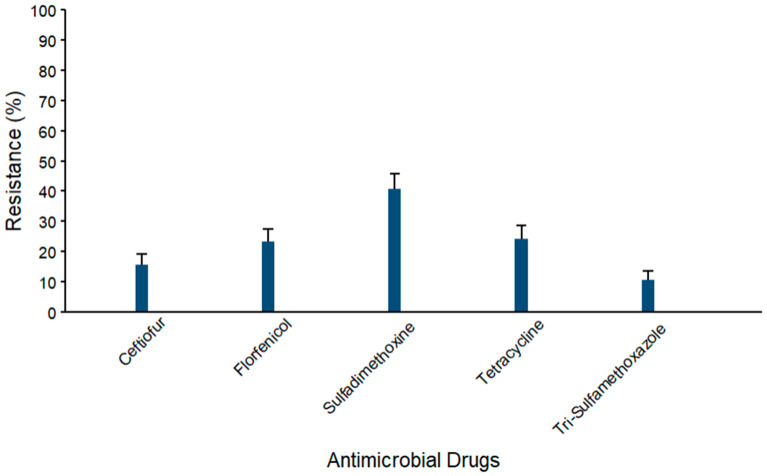
Percentages of waste milk samples with AMR in coliform species (*n* = 103). Error bars represent one standard error deviation from the mean. AMR of bacterial species against the AMD was determined by using MIC based on the CLSI cutoffs.

Results of AMR testing of all 348 isolates from the 100 WM samples collected on 8 of the original study dairies are presented here, while results of the 89 isolates from the 40 WM samples with AMD residue results are in the Supplementary Materials. Antimicrobial resistance profiles of the coliform isolates (*n* = 103) from WM samples are summarized in Figure 5. The maximum AMR prevalence was observed against sulfadimethoxine (40.8% ± 4.8), while mid-range AMR prevalence was observed against florfenicol (23.3% ± 4.2), tetracycline (24.3% ± 4.2), and ceftiofur (15.5% ± 3.6), and the lowest resistance against trimethoprim–sulfamethoxazole (10.7% ± 3.0).

**Figure 6:**
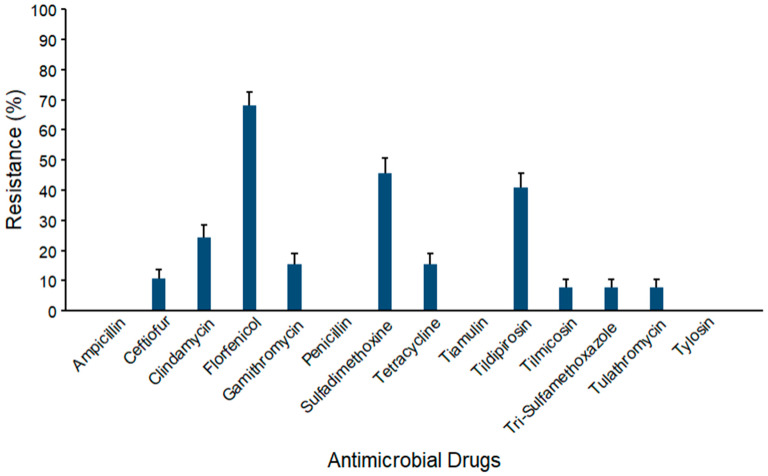
Percentages of waste milk samples with AMR in *Staphylococcus* spp. (*n* = 103). Error bars represent one standard error deviation from the mean. AMR of bacterial species against the AMD was determined by using MIC based on the CLSI cutoffs.

Prevalence estimates for AMR against *Staphylococcus* spp. (*n* = 103) are depicted in Figure 6. The maximum resistance was observed against florfenicol (68.0% ± 4.6). Mid-range AMR levels were observed for sulfadimethoxine (45.6% ± 4.9), tildipirosin (40.8% ± 4.8), clindamycin (24.3% ± 4.2), tetracycline (15.5% ± 3.6), ceftiofur (10.7% ± 3.0%), and gamithromycin (15.5% ± 3.6). Resistance levels below 10% were observed against tilmicosin (7.8% ± 2.6), trimethoprim–sulfamethoxazole (7.8% ± 2.6), and tulathromycin (7.8% ± 2.6). No resistance was detected against ampicillin and tiamulin.

**Figure 7:**
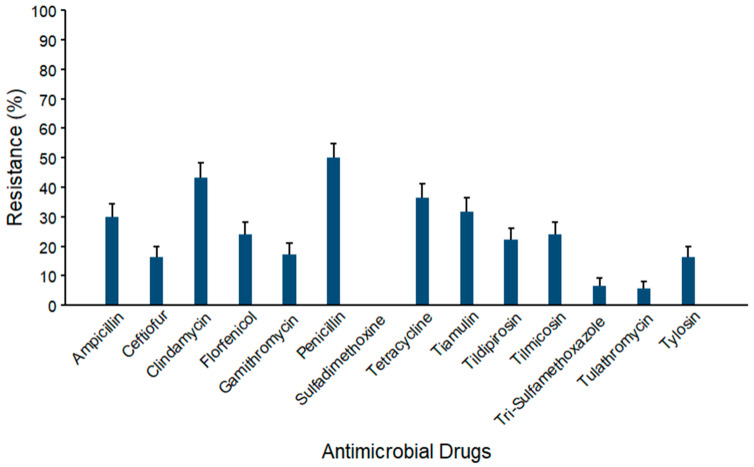
Percentages of waste milk samples with AMR in *Streptococcus* spp. (*n* = 104). Error bars represent one standard error deviation from the mean. AMR of bacterial species against the AMD was determined by using MIC based on the CLSI cutoffs.

Prevalence estimates for AMR against *Streptococcus* spp. (*n* = 104) are depicted in Figure 7. The maximum AMR prevalence was observed against penicillin (50.0% ± 4.9). Mid-range resistance levels were observed against clindamycin (43.3% ± 4.9), tetracycline (36.5% ± 4.7), tiamulin (31.7% ± 4.6), ampicillin (29.8% ± 4.5), tilmicosin (24.0% ± 4.2), florfenicol (24.0% ± 4.2), tildipirosin (22.1% ± 4.1), and gamithromycin (17.3% ± 3.7). The lowest two AMR prevalence estimates were against trimethoprim–sulfamethoxazole (6.7% ± 2.5) and tulathromycin (5.8% ± 2.3). No resistance was detected against sulfadimethoxine.

**Figure 8:**
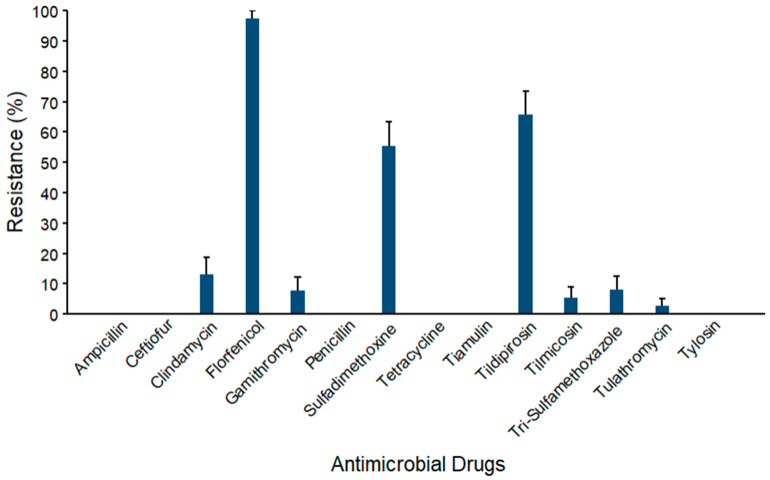
Percentages of waste milk samples with AMR in *S. aureus* (*n* = 38). Error bars represent one standard error deviation from the mean. AMR of bacterial species against the AMD was determined by using MIC based on the CLSI cutoffs.

Similarly, prevalence estimates for AMR against *S. aureus* (*n* = 38) are depicted in Figure 8. The maximum AMR prevalence was observed against florfenicol (97.4% ± 2.6). Furthermore, mid-range AMR prevalence was observed against tildipirosin (65.8% ± 7.7) and sulfadimethoxine (55.3% ± 8.1). The prevalences of AMR against clindamycin (13.2% ± 5.5), gamithromycin (7.9% ± 4.4), and trimethoprim–sulfamethoxazole (8.1% ± 4.5) were all below 15%. While the AMR prevalence estimate for tilmicosin was (5.3% ± 3.6), the lowest prevalence was observed for tulathromycin (2.6% ± 2.6). No resistance was documented against ampicillin, ceftiofur, penicillin, tetracycline, tiamulin, and tylosin.

#### 3.3.1. Regional Variation in AMR Among Isolates

**Figure 9:**
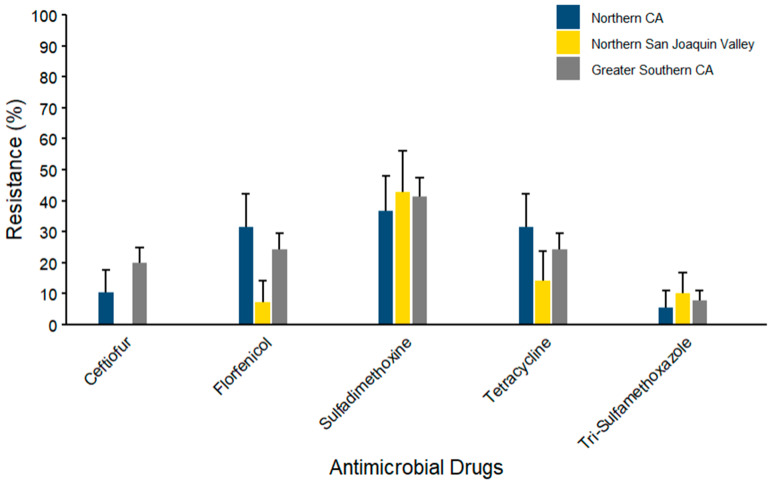
Antimicrobial resistance of coliform species isolated from waste milk samples collected from 8 California dairies by region (*n* = 103). Error bars represent one standard error deviation from the mean. AMR of bacterial species against the AMD was determined by using MIC based on the CLSI cutoffs.

Prevalences of antimicrobial resistance (AMR) profiles for coliform species (*n* = 103) isolated from WM samples across the regions of NCA, NSJV, and GSCA are summarized in Figure 9. The maximum percentage of resistance was observed against sulfadimethoxine (42.86% ± 13.23), with the maximum prevalence documented in the NSJV region, while mid-range resistance levels were observed against tetracycline (31.58% ± 10.66) and florfenicol (31.58% ± 10.66) in the NCA region. In contrast, the lowest resistance levels were observed against ceftiofur and trimethoprim–sulfamethoxazole across the three regions. No resistance was documented against ceftiofur in the NSJV region.

**Figure 10:**
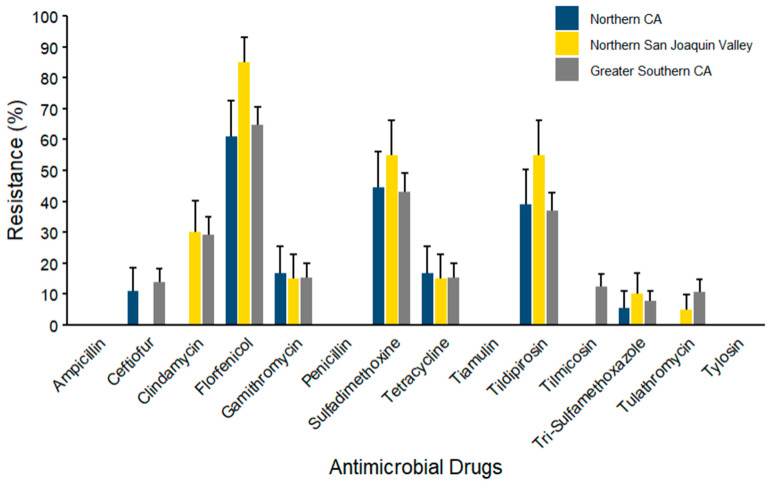
Resistance of *Staphylococcus* spp. isolated from waste milk samples collected from 8 California dairies by region (*n* = 103). Error bars represent one standard error deviation from the mean. AMR of bacterial species against the AMD was determined by using MIC based on the CLSI cutoffs.

For *Staphylococcus* isolates (*n* = 103) (Figure 10), the maximum AMR prevalence was exhibited against florfenicol (85.00% ± 7.98) in all three regions, with NSJV showing the greatest prevalence, followed by NCA and GSCA. Mid-range resistance levels were observed against tildipirosin (55.00% ± 11.12) and sulfadimethoxine (55.00% ± 11.12) across regions, with NSJV isolates again presenting the maximum percentages. Mid-range resistance levels were observed for clindamycin in two regions, while the lowest levels were noted for gamithromycin, tilmicosin, and trimethoprim–sulfamethoxazole. Ampicillin, ceftiofur, penicillin, tetracycline, tiamulin, and tylosin showed no detectable resistance in any of the three regions.

**Figure 11:**
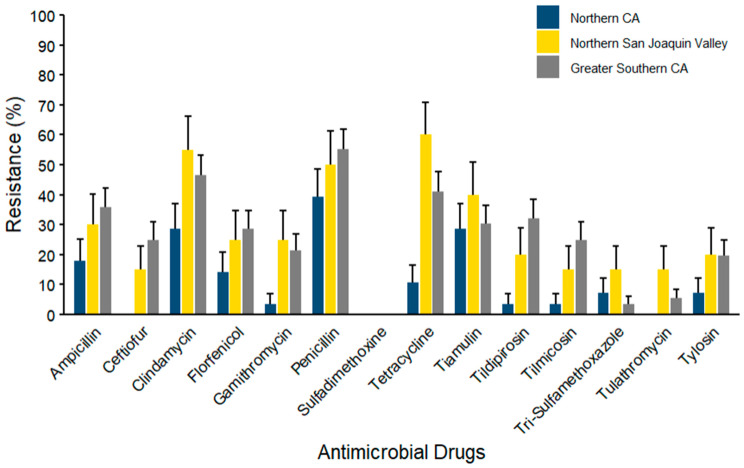
Resistance of *Streptococcus* spp. isolated from waste milk samples collected from 8 California dairies by region (*n* = 104). Error bars represent one standard error deviation from the mean. AMR of bacterial species against the AMD was determined by using MIC based on the CLSI cutoffs.

In *Streptococcus* spp. (*n* = 104) isolates (Figure 11), the maximum percentage of resistance was observed for tetracycline (60.00% ± 10.95) in NSJV, followed by clindamycin (55.00 ± 11.12) and penicillin (50.00% ± 11.18) in the same region. In the NCA and GSCA regions, high AMR prevalence was reported against penicillin (39.29% ± 9.23; 55.36% ± 6.64, respectively). Mid-range resistance was noted for ceftiofur, florfenicol, tiamulin, and tilmicosin across all regions, although the prevalence varied. Lower resistance percentages were detected for gamithromycin, trimethoprim–sulfamethoxazole, tulathromycin, and tylosin. No resistance was documented against sulfadimethoxine.

**Figure 12:**
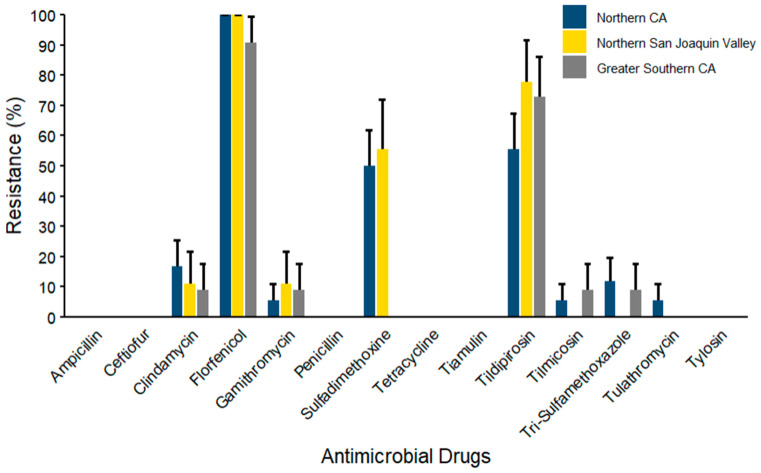
Resistance of *S. aureus* isolated from waste milk samples collected from 8 California dairies by region (*n* = 38). Error bars represent one standard error deviation from the mean. AMR of bacterial species against the AMD was determined by using MIC based on the CLSI cutoffs.

Finally, for *S. aureus* (*n* = 38) isolates (Figure 12), florfenicol resistance was the maximum across all three regions, with NCA and NSJV at 100% resistance and GSCA following closely at 90.91% and resistance against sulfadimethoxine (55.56% ± 16.56) and tildipirosin (77.78% ± 13.86) following next with resistance against Clindamycin in *S. aureus* observed at 16.67% ± 8.78. In contrast, lower AMR prevalence was observed against gamithromycin, tilmicosin, tulathromycin, and trimethoprim–sulfamethoxazole across the three regions. No resistance was documented against ampicillin, ceftiofur, penicillin, tetracycline, tiamulin, and tylosin in any of the three regions.

#### 3.3.2. Seasonal Variation in AMR Among Isolates

**Figure 13:**
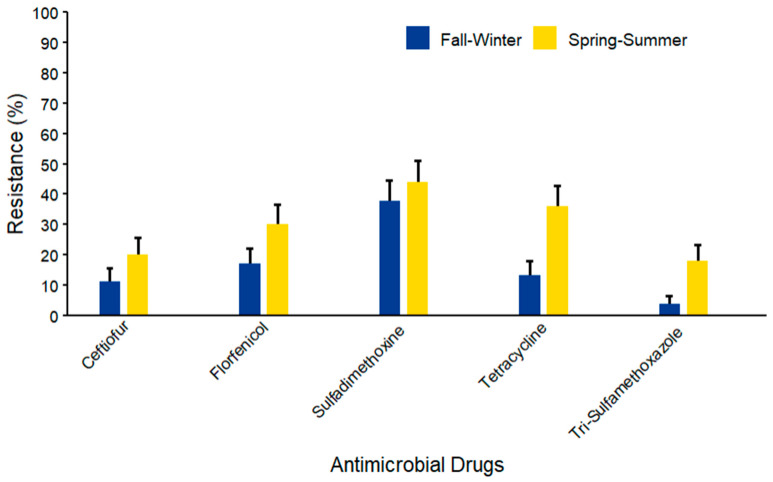
Antimicrobial resistance of coliform species (*n* = 103) isolated from waste milk samples collected from 8 California dairies by season. Error bars represent one standard error deviation from the mean. AMR of bacterial species against the AMD was determined by using MIC based on the CLSI cutoffs.

Considering seasonality, the prevalence of AMR among coliform species (*n* = 103) isolated from WM samples varied between fall–winter and spring–summer (Figure 13). Highest AMR prevalence was observed in the spring–summer season as compared to fall–winter against the five AMD. The maximum resistance was against sulfadimethoxine (44.00% ± 7.02) in the spring–summer season. Mid-range resistance was documented against tetracycline (36.00% ± 6.79) and florfenicol (30.00% ± 6.48) in the spring–summer season, while lower resistance levels were observed against ceftiofur (20.00% ± 5.66) and trimethoprim sulfamethoxazole (18.00% ± 5.43), with a consistent spring–summer increase.

**Figure 14:**
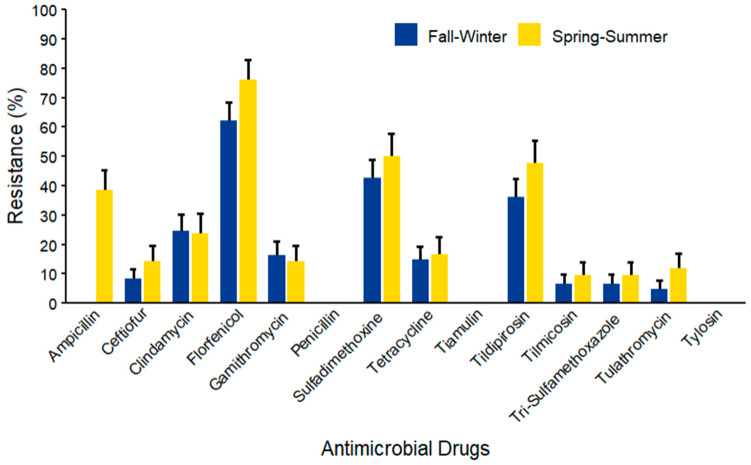
Antimicrobial resistance of *Staphylococcus* spp. (*n* = 103) isolated from waste milk samples collected from 8 California dairies by season. Error bars represent one standard error deviation from the mean. AMR of bacterial species against the AMD was determined by using MIC based on the CLSI cutoffs.

Among *Staphylococcus* spp. (*n* = 103), the maximum AMR prevalence was observed against florfenicol (76.19% ± 6.57) in both seasons (Figure 14). Mid-range resistance was observed for sulfadimethoxine (50.00% ± 7.72) and tildipirosin (47.62% ± 7.71), which were also higher in spring–summer compared to fall–winter. Lower resistance levels were observed against ampicillin, ceftiofur, clindamycin, gamithromycin, and tetracycline, with generally higher prevalence in the spring–summer season. Resistance levels against tilmicosin, trimethoprim–sulfamethoxazole, and tulathromycin fell below 10%, especially in the fall–winter season. However, no resistance was documented against penicillin, tiamulin, and tylosin.

**Figure 15:**
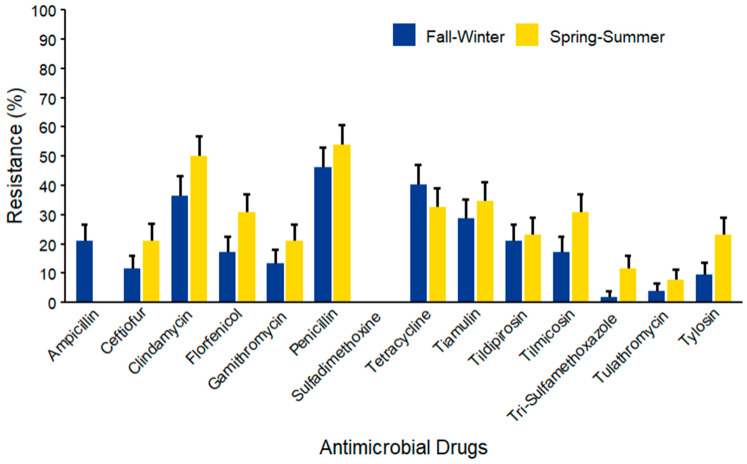
Antimicrobial resistance of *Streptococcus* spp. (*n* = 104) isolated from waste milk samples collected from 8 California dairies by season. Error bars represent one standard error deviation from the mean. AMR of bacterial species against the AMD was determined by using MIC based on the CLSI cutoffs.

For *Streptococcus* spp. isolates (*n* = 104) (Figure 15), the maximum AMR prevalence was recorded against penicillin (53.85% ± 6.91) in both seasons. Notably, high AMR prevalence was observed against tetracycline (40.38% ± 6.80) and clindamycin (50.00% ± 6.93) in fall–winter and spring–summer, respectively. Mid-range resistance levels were observed for ceftiofur and tiamulin, while lower resistance levels were observed against florfenicol, gamithromycin, tildipirosin, tilmicosin, and tylosin across both seasons. The lowest resistance was observed for trimethoprim–sulfamethoxazole and tulathromycin with minimal seasonal variations.

**Figure 16:**
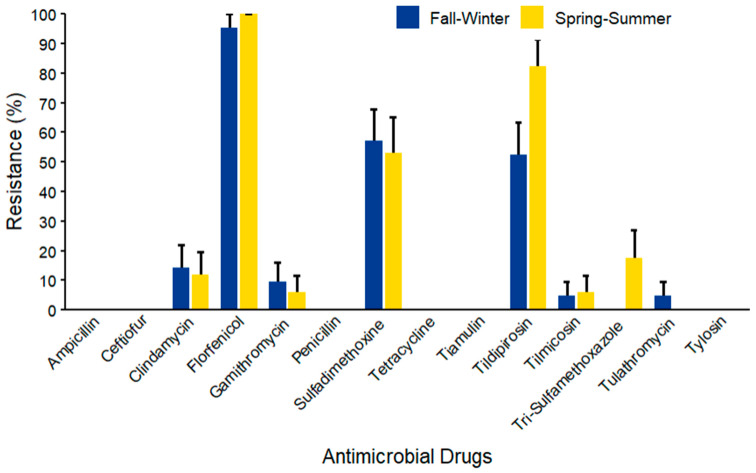
Antimicrobial resistance of *S. aureus* (*n* = 38) isolated from waste milk samples collected from 8 California dairies by season. Error bars represent one standard error deviation from the mean. AMR of bacterial species against the AMD was determined by using MIC based on the CLSI cutoffs.

In *S. aureus* (*n* = 38) isolates (Figure 16), the maximum AMR prevalence was observed against florfenicol in both seasons, with a 100% prevalence during spring–summer. Notably, mid-range AMR prevalence was observed against sulfadimethoxine (57.14% ± 10.80) and tildipirosin (82.35% ± 9.25) in fall–winter and spring–summer, respectively. No resistance was detected against ampicillin, ceftiofur, penicillin, tiamulin, and tylosin.

### 3.4. Interval-Censored Accelerated Failure Time (AFT) Survival Models for AMR

The final parametric interval-censored accelerated failure time (AFT) models for coliform species isolates are presented in Table 2. Results showed that there was no association between ceftiofur residue and AMR in coliforms (MIC ratio = 0.49; 95% CI: 0.06–3.71; *p* = 0.49). Sampling month was also associated with MIC variation: Higher MICs were observed against ceftiofur in sampling months April–June (MIC ratio = 10.68; 95% CI: 1.16–98.22; *p* = 0.04), while no significant changes were detected in months December–January, February–March, and July–August (*p* > 0.05).

For florfenicol, positive residue status was significantly associated with increased MIC (MIC ratio = 2.12; 95% CI: 1.44–3.10; *p* < 0.01). Sampling months December–January, February–March, and July–August showed significantly higher MICs (MIC ratios = 1.26, 2.00, and 2.00, respectively; all *p* < 0.01), whereas April–June was associated with a significant reduction in MIC (MIC ratio = 0.75; 95% CI: 0.67–0.84; *p* < 0.01). Regional differences were observed, with isolates from the GCSA region showing significantly lower MICs than those from the NCA (MIC ratio = 0.78; 95% CI: 0.60–0.99; *p* = 0.049). No significant difference was detected between NSJV and NCA (*p* = 0.92).

Tilmicosin and tetracycline were excluded from the analysis due to the absence of detectable residues. Penicillin was excluded because coliforms are intrinsically resistant, and the model for sulfadimethoxine did not converge due to multicollinearity and small sample size.

The final AFT model for *Streptococcus* spp. is shown in Table 3. The presence of ceftiofur residue was associated with more than tenfold increase in MIC compared to samples without detectable residue (MIC ratio = 10.51; 95% CI: 1.21–91.29; *p* = 0.03), indicating higher resistance levels. Tilmicosin and tetracycline were excluded due to the absence of detectable residues, and the models for sulfadimethoxine, florfenicol, and penicillin did not converge due to small sample size and multicollinearity.

For *Staphylococcus* spp., the final AFT model results are summarized in Table 4. Ceftiofur residue status was not significantly associated with resistance to it (MIC ratio = 0.55; 95% CI: 0.08–3.69; *p* = 0.54). Seasonal analysis showed no significant variation in MIC values, although spring–summer had a numerically higher MIC ratio compared to fall–winter (MIC ratio = 2.54; 95% CI: 0.58–11.07; *p* = 0.22). Tilmicosin and tetracycline were excluded due to the absence of detectable residues, and models for sulfadimethoxine, penicillin, and florfenicol did not converge due to small sample size.

The final AFT model for *S. aureus* (Table 5) indicated that ceftiofur residue status was not significantly associated with MIC (MIC ratio = 1.49; 95% CI: 0.62–3.56; *p* = 0.369). In addition to that, the region was a confounder to the association between residue and resistance as it showed >20% change in estimate; however, no significant regional differences were detected (*p* > 0.05). Tilmicosin and tetracycline were excluded due to the absence of detectable residues, and models for sulfadimethoxine, florfenicol, and penicillin did not converge due to small sample size.

## 4. Discussion

### 4.1. Antimicrobial Residues in WM

Waste milk is derived from hospital cows during AMD withdrawal periods or from recently calved cows transitioning from colostrum to milk, making the presence of AMD residues expected. Its composition, similar to that of whole milk, makes WM a low-cost nutrient source for calves. There are no AMD tolerance levels for calves, and although WM is not regulated by the FDA, we applied the FDA’s regulatory MRL intended for human consumption and evaluated its AMD residue content.

Waste milk samples collected over a 10-month period from a convenience sample of eight California dairies exceeded the FDA MRL for four AMDs. Ceftiofur was the most prevalent AMD residue found in 12 of the study’s 40 WM samples using ELISA testing, while penicillin, sulfadimethoxine, and florfenicol followed at 2 samples each out of 40. These findings show that feeding WM to calves on the study dairies may contain AMD concentrations that are known to exert selective pressure on bacteria over time [24,26]. A study done on controlled in vivo calf trials demonstrated that even sub-MIC residue concentrations can increase the percentage of resistant fecal E.coli, highlighting the selective-pressure interpretation without requiring residues to exceed MICs [18]. Our results are consistent with previous surveys of U.S. dairy farms reporting ceftiofur and cephapirin as among the most commonly detected AMD residues in WM [42]. Surveys of WM in the U.S. using LC-MS/MS confirm that beta-lactams, including ceftiofur, are commonly detected in pooled WM samples and can be detected at quantifiable concentrations, supporting the results we got in this current study [14,35]. Additionally, Tempini et al. (2018) have reported the detection of one AMD in 60% of dairy farms, with ceftiofur and oxytetracycline among the most prevalent residues [14]. Given the zero-milk withdrawal associated with parenteral ceftiofur administration in lactating cows, the presence of ceftiofur residues in the study WM samples is more consistent with its administration as an intramammary infusion for treatment of mastitis or as dry-off therapy than with systemic administration. These findings highlight mastitis and its treatment with intramammary infusions as an important source of these residues [42,43].

Florfenicol, an AMD used for bovine respiratory disease therapy in calves [4,14,44], has a zero-tolerance level in milk [45], as it is prohibited in lactating dairy cows and female dairy cattle older than 20 months, including dry cows. Nevertheless, florfenicol residues were detected in one WM sample from two of the eight study dairies, representing 2 of the 40 samples collected over the one-year period. Dairy records and survey questionnaires for the study herds do not include the use of florfenicol in female cattle > 20 months, regardless of lactating status. Therefore, the exact reason for florfenicol residues in 2 of the 40 WM samples is not known. This isolated finding suggests incidental exposure of lactating cows to florfenicol. Such exposure may be explained by extra-label drug use of florfenicol, or its use in non-lactating cattle younger than 20 months old, followed by environmental shedding of the drug in excreta and the subsequent exposure of lactating cows through recycled flush water. Supporting this hypothesis, florfenicol was detected in all the lagoon and recycled flush water samples collected from the current study dairies on the same days that WM samples were collected [46]. Additionally, AMR against florfenicol was observed in 96% of E.coli isolates in the original study’s fecal commensals isolated from 120 cows on the 10 study dairies, supporting recycled water exposure as a plausible pathway for farm-level florfenicol residue ecology even when adult-cow treatments are not recorded [15]. Other potential explanations may include individual animal-level factors that may have challenged the withdrawal period’s success in eliminating the AMD, such as improper dosage or route of administration. The latter explanations do not exclude potential for cross-contamination, diagnostic inaccuracies, or laboratory errors, although standard procedures, including proper sample collection and labeling methods, validated assay protocols, and use of positive and negative controls were followed, respectively. Further research is needed to confirm our findings related to the occurrence of florfenicol residues in WM and lagoon or recycled flush water on other dairies, in addition to outreach for antimicrobial stewardship.

Geographical and seasonal patterns were also observed in the current study. Notably, WM samples from the GSCA region had a higher prevalence of AMD residues, including ceftiofur, penicillin, and sulfadimethoxine, as compared to NCA and NSJV regions. In contrast, tetracycline did not exceed the allowable FDA MRL, and tilmicosin residues were not present in any region. These regional differences may be explained by herd management differences among the California dairies. Likewise, seasonal differences were minimal but suggested slightly higher residue occurrence in spring–summer than fall–winter for ceftiofur. Such variation may be attributable to differences in disease incidence, treatment practices related to herd size, mastitis management strategies, or seasonal increases in AMD use associated with higher disease incidence during the summer. The original study done on lagoon samples supported the fact that regional and seasonal comparisons can be underpowered when there is a farm difference in management and sampling intensity [46]. However, further investigation is required to confirm and better characterize these regional and seasonal trends.

### 4.2. AMR Profiles Among Isolates

Resistance prevalence ranged from 5% to 97% across bacterial species and against the antimicrobial classes, indicating a variability in AMR profiles among isolates. We examined four groups of bacteria (coliforms, *Streptococcus* spp., *Staphylococcus* spp., and *S. aureus*) and found resistance against several antimicrobial classes in each group (Figure 5, Figure 6, Figure 7 and Figure 8, Tables S3–S6). The maximum AMR prevalence in coliform isolates was observed against sulfadimethoxine (40.8%). Wm surveys that included bacterial cultures routinely recovered *Streptococcus* spp. and *Staphylococcus* spp. at high frequency, often greater than 80%, with *E. coli* also common, indicating that it matches the current study’s WM matrices [14].

The AMR profiles in this study may be influenced by historical antimicrobial exposure on the dairies, as reported in a previous USDA study, where 8 out of 10 dairies treated 85.4% of mastitis using AMDs [47]. Dairy calf-rearing and WM environments often harbor commensal *E. coli* and other bacteria that have acquired resistance to commonly used AMDs, such as tetracyclines and sulfonamides [24,48,49]. In our study, even though tetracycline residues did not exceed the FDA-established MRL, about 24% of isolates showed phenotypic resistance to tetracycline. This finding is consistent with reports indicating that tetracycline resistance genes are ubiquitous in dairy farm microbiota, likely due to both historical and ongoing use of oxytetracycline in cattle [24,48,49]. Co-resistance was also evident in our staphylococcal isolates; notably, *S. aureus* isolates demonstrated a resistance prevalence of 97.4% to florfenicol and 65.8% to tildipirosin. This might suggest the presence of mobile genetic elements such as the chloramphenicol–florfenicol resistance (*cfr*) gene, which confers cross-resistance to phenicol, lincosamides, pleuromutilin, and macrolides in *Staphylococci* [50,51]. Molecular studies also demonstrate that cfr can be carried on plasmids in animal-origin staphylococci and may be a determinant factor to mobile phenicol resistance and cross-class resistance patterns in *Staphylococcus* species [52]. In fact, co-selection of resistance genes is a well-recognized phenomenon in farm environments: Use of one antimicrobial can maintain resistance to another class of AMD if the genes are linked [53]. For instance, calves fed milk containing β-lactam antimicrobials have been shown to shed *E. coli* that are florfenicol-resistant at higher rates than calves fed no-antibiotic milk [24]. *Streptococcus* spp. isolates remained susceptible to sulfadimethoxine with no resistance reported and had lower resistance to macrolides like tulathromycin and trimethoprim–sulfamethoxazole.

The current study also identified notable regional and seasonal influences on AMR prevalence. Among coliform species, the regional patterns of AMR prevalence varied significantly. Regarding seasonal patterns, all bacterial species’ isolates showed a higher AMR prevalence in the spring–summer season as compared to the fall–winter season. In a study of raw milk in China, coliform species were isolated with a higher degree of incidence during the spring–summer season and showed higher AMR prevalence to sulfonamides and beta-lactams, while lower resistance profiles were reported on tetracyclines and macrolides [54], which correlates with our current study. However, these findings contrast with reports indicating that mastitis incidence tends to increase during the fall–winter season, leading to higher AMR prevalence in dairies, as observed in fecal commensal *E. coli* isolates [31]. In the current study, a tenfold increase in MICs for ceftiofur was observed during April–June compared to October–November (*p* = 0.04). These temporal effects did not follow a consistent seasonal pattern and may reflect management effects. Regionally, coliforms from GSCA had lower AMR against florfenicol compared to those from NCA (22% reduction, *p* = 0.049), because these two WM samples were collected from NCA and NSJV.

### 4.3. Association Between AMD Residues and AMR Prevalence Among Isolates

In the current study, we compared MICs of isolates from residue-positive and residue-free samples while adjusting for confounders. For coliforms, florfenicol residues were linked to more than a two-fold increase in MIC (MIC ratio = 2.12, *p* < 0.01), indicating significantly higher resistance in residue-positive samples. These findings align with prior findings in calves from two studies that reported that calves fed WM had a higher prevalence of florfenicol-resistant *E. coli* (at 6 weeks old) compared to calves fed AMD-free milk replacers [24,29]. Langford et al. (2003) [8] also demonstrated a clear dose–response whereby higher antimicrobial levels in WM fed to calves led to greater proportions of resistant gut *E. coli*. Likewise, Pereira et al. (2014) [18] showed in a study that calves fed milk spiked with lower concentrations of AMD rapidly developed a higher prevalence of antimicrobial-resistant *E. coli* in their feces compared to calves that were fed AMD-free milk. These results reinforce that florfenicol residues in WM exert selective pressure on coliform populations.

For ceftiofur, no significant association was observed between its residue and resistance in coliforms (MIC ratio = 0.49, *p* = 0.49). Despite the point-estimate suggesting lower MICs in residue-positive samples, the small sample size (*n* = 18) meant a wide confidence interval and lack of significance. Similar mixed outcomes have been reported in WM-fed calves, where resistance initially increases but later declines as susceptible strains re-colonize [24]. Maynou et al. (2017) [29] found that WM feeding increased cephalosporin-resistant *E. coli*.

Analysis of *Streptococcus* spp. isolates (*n* = 25) showed a strong association between ceftiofur residues in WM and resistance, with residue-positive isolates displaying over a 10-fold higher MIC (MIC ratio = 10.51, *p* = 0.03). This indicates that ceftiofur exposure selects for resistant *Streptococcus* spp. contributing to higher AMR prevalence [4,26]. *Streptococcus* spp. is frequently isolated and found in WM, reported in up to 84% of samples [14], as *Streptococcus* spp. can cause mastitis.

For *Staphylococcus* spp., no significant association was found between AMD residues in WM and MICs in our AFT model. Ceftiofur was the only analyzable residue, but isolates from ceftiofur-positive samples did not differ significantly from residue-free ones (MIC ratio = 0.55, *p* = 0.54). This result could be due to several factors, including the small sample size (*n* = 28) failing to detect significant effects. Ceftiofur is primarily used against Gram-negative infections and some *Streptococcus*; it is not the first-line choice for staphylococcal mastitis [55]. Differences between spring–summer and fall–winter were not significant (MIC ratio = 2.54, *p* = 0.22).

Finally, *S. aureus* was analyzed separately due to its role as a contagious mastitis pathogen and distinct behavior from other staphylococci. In our model (*n* = 18 isolates), no significant association was found between ceftiofur residues and *S. aureus* MICs (MIC ratio = 1.49, *p* = 0.369), nor were regional differences detected (NSJV *p* = 0.92; GSCA *p* = 0.50 vs. NCA). However, regions confounded the relationship as evidenced by >20% change in estimate method. Notably, many *S. aureus* strains produce penicillinase, making them penicillin-resistant regardless of residue exposure [56,57], though ceftiofur remains effective against non-MRSA strains [55,58]. Our findings suggest WM with ceftiofur residues did not increase ceftiofur-resistant *S. aureus*, possibly due to very low residue levels, or given that ceftiofur is not commonly used for *S. aureus* mastitis, a contagious mastitis commonly controlled by culling. Overall, the current study indicates that ceftiofur in WM does not significantly impact *S. aureus* resistance, though existing research remains limited and focused on its epidemiology rather than WM-associated resistance.

A limitation of this study is the relatively small number of herds sampled, which may limit the representativeness of the findings across California dairies and reduce statistical power to detect associations between antimicrobial residues (AMDs) and AMR prevalence. Combining ELISA screening results with Liquid Chromatography/Mass Spectrometry (LC/MS) confirmation could provide more robust insights into residue detection, while screening a broader range of AMDs would strengthen the ability to evaluate associations with commonly used AMDs. In addition, incorporating genotype testing of resistance determinants, including AMR genes and mobile genetic elements, could clarify mechanisms of resistance and potential cross-resistance, helping to confirm whether the observed phenotypic patterns are directly linked to residue exposure. Finally, although region and sampling month were accounted for in the statistical models, unmeasured management or environmental variables may still have influenced both residue presence and AMR, which could affect the generalizability of the findings beyond the studied farms.

## 5. Conclusions

The current research focused on evaluating AMD residues in WM samples, and results showed that a substantial number of WM samples contained detectable levels of AMD residues. Ceftiofur was the most prevalent, followed by florfenicol, penicillin, and sulfadimethoxine. Tilmicosin was absent, and tetracycline residues were within the allowed FDA-established MRL. Analysis showed regional and seasonal variations among residues and their presence in WM. AFT models revealed significant associations between AMD residues and increased resistance. No statistically significant associations were found between AMD residues and AMR prevalence in *Staphylococcus* spp. or *S. aureus*. Results showed robust associations for florfenicol in coliforms and ceftiofur in *Streptococcus* spp. We anticipate that the current findings contribute to the current understanding of AMD residues in WM on dairies and provide directions for future research on mechanisms of acquisition of such resistance. Increased frequency of sampling and inclusion of larger sample sizes will assist in characterizing the resistance of WM and its association with bacterial populations.

## Figures and Tables

**Table 1:** Percentage of waste milk samples testing positive for antimicrobial residues.

Antimicrobial Drugs	Kit Detection Limit ^1^		FDA MRL ^2^		Combined Metric ^3^	
	Estimate% (SE)	95% CI	Estimate% (SE)	95% CI	Estimate% (SE)	95% CI
Penicillin	62.5 (7.75)	46.1, 76.5	5.0 (3.49)	1.2, 18.9	5.0 (3.49)	1.2, 18.9
Ceftiofur	62.5 (7.75)	46.1, 76.5	30.0 (7.24)	17.6, 46.3	30.0 (7.24)	17.6, 46.3
Sulfadimethoxine	22.5 (6.69)	11.8, 38.7	5.0 (3.49)	1.2, 18.9	5.0 (3.49)	1.2, 18.9
Tetracycline ^4^	40.0 (7.84)	25.6, 56.4	0 (0.0)	-	0 (0.0)	-
Florfenicol	5.0 (3.49)	1.2, 18.9	5.0 (3.49)	1.2, 18.9	5.0 (3.49)	1.2, 18.9
Tilmicosin	0 (0.0)	-	0 (0.0)	-	0 (0.0)	-

^1^ Kit detection limit is the lowest concentration of an AMD that the ELISA kit can reliably detect in WM samples. ^2^ FDA MRL—the maximum legally permitted concentration of a specific AMD residue in milk, as defined by the U.S. Food and Drug Administration. ^3^ Combined Metric—the final classification based on FDA MRL coupled with the ELISA kit detection thresholds to account for the limitations of the kit, particularly when residue levels fell below the kit’s detection limit. There were no samples that were below the kit detection limit but at or greater than the FDA MRL, rendering residue interpretation across the FDA and combined metrics identical. ^4^ Tetracycline was within the allowable limit from all samples based on the FDA-established maximum residue limit of 300 ppb.

**Table 2:** Final parametric interval-censored accelerated failure time survival models evaluating the association between antimicrobial residues and AMR in coliform species (*n* = 18).

Antimicrobial Drug	Variables	Levels	Coefficient	SE **	MIC Ratio	95% CI **	*p*-Value
Ceftiofur	Residue	Negative	Referent	—	—	—	—
		Positive	−0.72	0.506	0.49	[0.06, 3.71]	0.49
	Sampling month *	October–November	Referent	—	—	—	—
		December–January	0.57	1.773	1.77	[0.25, 12.63]	0.57
		February–March	−6.87	0.004	0.001	[0, 4.04]	0.10
		April–June	2.37	12.09	10.68	[1.16, 98.22]	0.04
		July–August	−0.09	0.398	0.91	[0.39, 2.14]	0.83
	Intercept	—	−1.31	0.068	0.27	[0.17, 0.44]	<0.01
Florfenicol	Residue	Negative	Referent	—	—	—	—
		Positive	0.75	0.412	2.12	[1.44, 3.10]	<0.01
	Sampling month *	October–November	Referent	—	—	—	—
		December–January	0.23	0.075	1.26	[1.12, 1.41]	<0.01
		February–March	0.69	0.043	2.00	[1.92, 2.09]	<0.01
		April–June	−0.29	0.043	0.75	[0.67, 0.84]	<0.01
		July–August	0.69	0.064	2.00	[1.88, 2.13]	<0.01
	Region *	NCA	Referent	—	—	—	—
		NSJV	−0.01	0.106	0.99	[0.80, 1.22]	0.92
		GCSA	−0.25	0.099	0.78	[0.60, 0.99]	0.049
	Intercept	—	0.94	0.339	2.55	[1.96, 3.31]	<0.01

* Confounder based on >20% change in point-estimate. ** SE and 95% CI were computed for the MIC ratio.

**Table 3:** Final parametric interval-censored accelerated failure time survival models evaluating the association between antimicrobial residues and AMR in *Streptococcus species* (*n* = 25).

Antimicrobial Drug	Variables	Levels	Coefficient	SE **	MIC Ratio	95% CI **	*p*-Value
Ceftiofur	Residue	Negative	Referent	—	—	—	—
		Positive	2.35	11.59	10.51	[1.21, 91.29]	0.03
	Intercept	—	−5.70	0.838	0.003	[0, 0.37]	0.02

** SE and 95% CI were computed for the MIC ratio.

**Table 4:** Final parametric interval-censored accelerated failure time models evaluating the association between antimicrobial residues and AMR in *Staphylococcus* spp. (*n* = 28).

Antimicrobial Drug	Variables	Levels	Coefficient	SE **	MIC Ratio	95% CI **	*p*-Value
Ceftiofur	Residue	Negative	Referent	—	—	—	—
		Positive	−0.59	0.536	0.55	[0.08, 3.69]	0.54
	Season *	Fall–Winter	Referent	—	—	—	—
		Spring–Summer	0.93	1.907	2.54	[0.58, 11.07]	0.22
	Intercept	—	−0.87	0.183	0.42	[0.18, 0.99]	0.046

* Confounder based on >20% change in point-estimate. ** SE and 95% CI were computed for the MIC ratio.

**Table 5:** Final parametric interval-censored accelerated failure time survival models evaluating the association between antimicrobial residues and AMR in *S. aureus* (*n* = 18).

Antimicrobial Drug	Variables	Levels	Coefficient	SE **	MIC Ratio	95% CI **	*p*-Value
Ceftiofur	Residue	Negative	Referent	—	—	—	—
		Positive	0.39	0.663	1.49	[0.62, 3.56]	0.369
	Region *	NCA	Referent	—	—	—	—
		NSJV	0.02	0.253	1.02	[0.63, 1.66]	0.92
		GSCA	−0.37	0.379	0.69	[0.23, 2.02]	0.50
	Intercept	—	0.26	0.129	0.77	[0.56, 1.08]	<0.01

* Confounder based on >20% change in point-estimate. ** SE and 95% CI were computed for the MIC ratio.

## Data Availability

This study was sponsored by the California Department of Food and Agriculture and is subject to California Food and Agriculture Code (FAC) Sections 14400 to 14408. FAC Section 14407 requires that data collected be held confidential to prevent individual identification of a farm or business; as such, raw data from this study cannot be shared.

## Supplementary Materials

The following supporting information can be downloaded at: https://www.mdpi.com/article/10.3390/microorganisms14030620/s1. Table S1: Cutoff thresholds used to determine the presence of antimicrobial drug (AMD) residues in WM Samples. The residue levels were estimated using ELISA. The Kit Detection Limits values were provided in the manufacturer’s guidelines, while FDA Maximum Residue Limits are regulatory standards. All cutoff values are measured in parts per billion (ppb); Figure S1: Percentages of waste milk samples with antimicrobial drug (AMD) residue by sampling point based on the combined metric. Error bars represent one standard error deviation from the mean; Table S2: Percentage of antimicrobial drug (AMD) residues detected in waste milk samples collected from 8 California dairies (*n* = 40) based on the combined metric of FDA’s MRL and the ELISA kit detection thresholds, stratified by region (NCA, NSJV, GSCA) and season (Fall-winter, Spring-Summer); Table S3: Frequency of Coliform spp. isolated from waste milk samples by AMR testing as measured by minimum inhibitory concentration (µg/mL). Waste milk samples were collected from 8 California Dairies from Oct 2018–Aug 2019 (*n* = 103); Table S4: Frequency of *Staphylococcus* spp. isolated from waste milk samples by AMR testing as measured by minimum inhibitory concentration (µg/mL). Waste milk samples were collected from 8 California Dairies from Oct 2018–Aug 2019 (*n* = 103); Table S5: Frequency of *Streptococcus* spp. isolated from waste milk samples by AMR testing as measured by minimum inhibitory concentration (µg/mL). Waste milk samples were collected from 8 California Dairies from Oct 2018–Aug 2019 (*n* = 104); Table S6: Frequency of *S. aureus* isolated from waste milk samples by AMR testing as measured by minimum inhibitory concentration (µg/mL). Waste milk samples were collected from 8 California Dairies from Oct 2018–Aug 2019 (*n* = 38).
