# Supplementary material for: Epidemiology of Antimicrobial Residues and Phenotypic Resistance of Bacterial Isolates from Waste Milk on California Dairies
Source: Microorganisms. 2026 Mar 10;14(3):620. doi: 10.3390/microorganisms14030620 (PMC13029155; doi:10.3390/microorganisms14030620)
Supplement: Supplementary file 1 [file microorganisms-14-00620-s001.zip › microorganisms-4159608-supplementary.pdf]

Table S1. Cutoff thresholds used to determine the presence of antimicrobial drug (AMD) residues in WM Samples. The residue levels were estimated using ELISA. The Kit Detection Limits values were provided in the manufacturer's guidelines, while FDA Maximum Residue Limits are regulatory standards. All cutoff values are measured in parts per billion (ppb).

**Antimicrobial Drug**	**Cut Off points**	
	**Kit Detection Limit (ppb[1])**	**FDA MRL (ppb)**
Penicillin	2	5
Ceftiofur	5	100
Sulfadimethoxine	5	10
Tetracycline	3	300
Florfenicol	0.5	0
Tilmicosin	5	None[2]

[1]ppb – parts per billion (unit of measurement)

[2]FDA MRL for the AMD Tilmicosin have not been established.

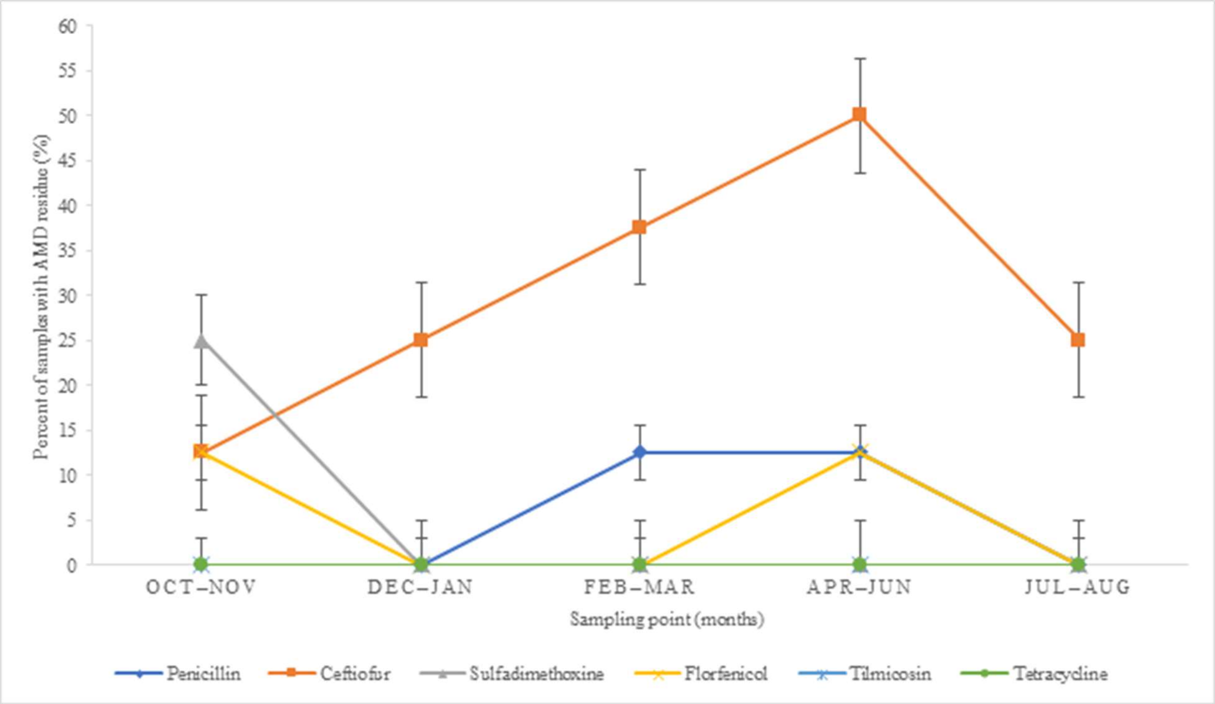

Figure S1. Percentages of waste milk samples with antimicrobial drug (AMD) residue by sampling point based on the combined metric. Error bars represent one standard error deviation from the mean.

Table S2. Percentage of antimicrobial drug (AMD) residues detected in waste milk samples collected from 8 California dairies (n = 40) based on the combined metric of FDA's MRL and the ELISA kit detection thresholds, stratified by region (NCA, NSJV, GSCA) and season (Fall-winter, Spring-Summer).

Antimicrobial Drug	Region [N=40] % (n[1]; SE[2]; LL[3], UL[4])			Season [N=40] % (n[1]; SE[2]; LL[3], UL[4])	
	NCA (N=15)	NSJV (N=10)	GSCA (N=15)	Fall-winter (N=24)	Spring-Summer (N=16)
Penicillin	0 (-; -; –)	0 (-; - ; –)	13.3 (2; 8.8; 3.2, 41.7)	4.2 (1; 4.1; 0.5, 25.6)	6.3 (1; 6.1; 0.8, 35.0)
Ceftiofur	0 (-; -; –)	30.0 (3; 14.5; 9.6, 63.4)	60.0 (9; 12.7; 34.1, 81.3)	25.0 (6; 8.8; 11.4, 46.4)	37.5 (6; 12.1; 17.4, 63.0)
Sulfadimethoxine	0 (-; -; –)	0 (-; - ; –)	13.3 (2; 8.8; 3.2, 41.7)	8.3 (2; 5.6; 2.0, 28.8)	0 (-; -; –)
Tetracycline	0 (-; -; –)	0 (-; - ; –)	0 (-; -; –)	0 (- ; -; –)	0 (-; -; –)
Florfenicol	13.3 (2; 8.8; 3.2, 41.7)	0 (-; -; –)	0 (-; -; –)	4.2 (1; 4.1; 0.5, 25.6)	6.3 (1; 6.1; 0.8, 35.0)
Tilmicosin	0 (-; -; –)	0 (-; - ; –)	0 (-; -; –)	0 (- ; -; –)	0 (-; -; –)

*NCA = Northern California Area, NSJV = North San Joaquin Valley, GSCA = Greater Southern California Valley
N is the total number of WM samples collected from the representative 8 farms and from the three regions and the two sampling seasons.

[1]n is the total number of positive samples obtained.

[2]SE = Standard Deviation

[3]LL = Lower limit of 95% Confidence Interval.

[4]UL = Lower limit of 95% Confidence Interval.

Table S3. Frequency of Coliform spp. isolated from waste milk samples by AMR testing as measured by minimum inhibitory concentration (μg/ml). Waste milk samples were collected from 8 California Dairies from Oct 2018 - Aug 2019 (n = 103)

Antimicrobial Drugs	Frequency and percentage of isolates with MICs (μg/ml)[1]													Resistance %	MIC 50	MIC 90
	≤0.12	0.25	0.5	1	2	4	8	16	32	64	128	256	512			
Ceftiofur		27 (26.21)	44 (42.72)	12 (11.65)	4 (3.88)	**3 (2.91)**	13 (12.62)							15.53	0.5	8
Florfenicol		1 (0.97)	3 (2.91)	4 (3.88)	18 (17.48)	53 (51.46)	**24 (23.3)**							23.3	4	8
Sulfadimethoxine												61 (59.22)	**42 (40.78)**	40.78	256	512
Tetracycline			10 (9.71)	28 (27.18)	36 (34.95)	4 (3.88)	**25 (24.27)**							24.27	2	8
Trimethoprim-Sulfamethoxazole					92 (89.32)	**11 (10.68)**								10.68	2	4

1. Bold and Underlined cells determine the MIC resistance breakpoints.

Table S4. Frequency of *Staphylococcus* spp. isolated from waste milk samples by AMR testing as measured by minimum inhibitory concentration (µg/ml). Waste milk samples were collected from 8 California Dairies from Oct 2018 - Aug 2019 (n = 103)

**Antimicrobial Drugs**	**Frequency and percentage of isolates with MICs (µg/ml)[1]**													**Resistance[2] %**	**MIC 50**	**MIC 90**
	**≤0.12**	**0.25**	**0.5**	**1**	**2**	**4**	**8**	**16**	**32**	**64**	**128**	**256**	**512**			
Ampicillin		4 (22.22)	3 (16.67)	3 (16.67)	2 (11.11)	2 (11.11)	2 (11.11)	2 (11.11)						-	1	16
Ceftiofur		16 (15.53)	25 (24.27)	30 (29.13)	21 (20.39)	**7 (6.80)**	4 (3.88)							10.68	1	4
Clindamycin		64 (62.14)	14 (13.59)	**10 (9.71)**	4 (3.88)	2 (1.94)	2 (1.94)	7 (6.79)						24.26	0.25	4
Florfenicol		0 (0)	0 (0)	5 (4.85)	28 (27.18)	**58 (56.31)**	12 (11.65)							67.96	4	8
Gamithromycin				76 (73.79)	7 (6.80)	4 (3.88)	**16 (15.53)**							15.53	1	8
Penicillin		4 (22.22)	1 (5.56)	2 (11.11)	3 (16.67)	0 (0)	8 (44.45)							-	2	8
Sulfadimethoxine												56 (54.37)	**47 (45.63)**	45.63	256	512
Tetracycline			76 (73.79)	9 (8.74)	2 (1.94)	0 (0)	**16 (15.53)**							15.53	0.5	8
Tiamulin			20 (19.42)	27 (26.21)	5 (4.85)	3 (2.91)	3 (2.91)	2 (1.94)	43 (41.74)					-	2	32
Tildipirosin				8 (7.77)	24 (23.3)	29 (28.16)	**25 (24.27)**	17 (16.51)						40.78	4	16
Tilmicosin					81 (78.64)	11 (10.68)	3 (2.91)	**8 (7.76)**						7.76	2	8
Trimethoprim-Sulfamethoxazole					95 (92.23)	**8 (7.77)**								7.77	2	2

Tulathromycin					91 (88.35)	4 (3.88)	**3 (2.91)**	5 (4.85)				7.76	8	16
Tylosin	20 (19.42)	31 (30.10)	40 (38.83)	3 (2.91)	1 (0.97)	2 (1.94)	6 (5.83)					-	2	4

[1] Bold and Underlined cells determine the MIC resistance breakpoints.

[2] Resistance breakpoints are missing for ampicillin, penicillin, tiamulin and tylosin.

Table S5. Frequency of *Streptococcus* spp. isolated from waste milk samples by AMR testing as measured by minimum inhibitory concentration (µg/ml). Waste milk samples were collected from 8 California Dairies from Oct 2018 - Aug 2019 (n = 104)

Antimicrobial Drugs	Frequency and percentage of isolates with MICs (µg/ml)[1]													Resistance[2] %	MIC 50	MIC 90
	≤0.12	0.25	0.5	1	2	4	8	16	32	64	128	256	512			
Ampicillin		73 (70.19)	**12 (11.54)**	9 (8.65)	4 (3.85)	1 (0.96)	0 (0)	5 (4.81)						29.81	0.25	2
Ceftiofur		65 (62.50)	13 (12.50)	7 (6.73)	2 (1.92)	**3 (2.88)**	14 (13.46)							16.34	0.25	8
Florfenicol		0 (0)	0 (0)	16 (15.38)	63 (60.58)	**19 (18.27)**	6 (5.76)							24.03	2	4
Gamithromycin				83 (79.81)	0 (0)	3 (2.88)	**18 (17.31)**							17.31	1	8
Penicillin	52 (50)	**17 (16.35)**	10 (9.62)	7 (6.73)	3 (2.88)	6 (5.77)	9 (8.65)	8 (7.69)						50	0.25	4
Sulfadimethoxine												25 (24.04)	79 (75.96)	-	512	512
Tetracycline			48 (46.15)	8 (7.69)	10 (9.62)	**5 (4.81)**	33 (31.73)							21.15	1	8
Tiamulin			43 (41.35)	6 (5.77)	8 (7.69)	7 (6.73)	5 (4.81)	2 (1.92)	**33 (31.73)**					31.73	2	32
Tildipirosin				22 (21.15)	20 (19.23)	39 (37.5)	**6 (5.77)**	17 (16.34)						22.11	4	16
Tilmicosin					29 (27.88)	22 (21.15)	28 (26.92)	**25 (24.04)**						24.04	8	16
Trimethoprim-Sulfamethoxazole					97 (93.27)	**7 (6.73)**								6.73	2	2
Tulathromycin							94 (90.38)	4 (3.85)	**0 (0)**	6 (5.77)				5.77	8	8

Tylosin	51 (49.04)	27 (25.96)	5 (4.81)	3 (2.88)	1 (0.96)	<u>**3 (2.88)**</u>	14 (13.47)	10 (9.62)				16.35	1	64

[1] Bold and Underlined cells determine the MIC resistance breakpoints.

[2] Resistance breakpoints are missing for sulfadimethoxine.

Table S6. Frequency of *S. aureus* isolated from waste milk samples by AMR testing as measured by minimum inhibitory concentration (µg/ml). Waste milk samples were collected from 8 California Dairies from Oct 2018 - Aug 2019 (n = 38)

Antimicrobial Drug	Frequency and percentage of isolates with MICs (µg/ml)[1]													Resistance[2] %	MIC 50	MIC 90
	≤0.12	0.25	0.5	1	2	4	8	16	32	64	128	256	512			
Ampicillin		0 (0)	0 (0)	0 (0)	1 (100)	0 (0)	0 (0)	0 (0)						-	2	2
Ceftiofur		0 (0)	7 (18.42)	27 (71.05)	4 (10.53)	0 (0)	0 (0)							-	1	2
Clindamycin		29 (76.32)	4 (10.53)	2 (5.26)	0 (0)	**1 (2.63)**	0 (0)	0 (0)	2 (5.26)					7.89	0.25	2
Florfenicol		0 (0)	0 (0)	0 (0)	1 (2.63)	**34 (89.47)**	3 (7.89)	0 (0)						97.36	4	8
Gamithromycin				34 (89.47)	1 (2.63)	0 (0)	**1 (2.63)**	2 (5.26)						7.89	1	4
Penicillin								1 (100)						-	16	16
Sulfadimethoxine												17 (44.74)	**21 (55.26)**	55.26	512	512
Tetracycline			33 (86.84)	4 (10.53)	1 (2.63)									-	0.5	1
Tiamulin			19 (50)	13 (34.21)	0 (0)	0 (0)	0 (0)	2 (5.26)	0 (0)	4 (10.53)				-	1	64
Tildipirosin					2 (5.26)	11 (28.95)	**22 (57.89)**	1 (2.63)	2 (5.26)					65.78	8	16
Tilmicosin					36 (94.74)	0 (0)	0 (0)	**0 (0)**	2 (5.26)					5.26	2	2

Trimethoprim-Sulfamethoxazole			36 (94.74)	**2 (5.26)**						5.26	2	2
Tulathromycin					35 (92.11)	2 (5.26)	0 (0)	**0 (0)**	1 (2.63)	2.63	8	16
Tylosin	1 (2.63)	22 (57.89)	13 (34.21)					2 (5.26)		-	1	2

[1] Bold and Underlined cells determine the MIC resistance breakpoints.

[2] Resistance breakpoints are missing for tetracycline, tiamulin, penicillin, ampicillin, ceftiofur and sulfadimethoxine.
